# Healthcare professionals’ practices and barriers in assessing and promoting physical activity in primary care: a descriptive study

**DOI:** 10.1186/s12875-025-03138-9

**Published:** 2025-12-22

**Authors:** Geneviève Laflamme, François Trudeau, Marie-Claude Lehoux, Jean Lemoyne, Magali Brousseau-Foley, Julie Houle

**Affiliations:** 1 Department of Human Kinetics, Université du Québec à Trois-Rivières, Trois- Rivières, Québec Canada; 2 Infrastructure de recherche en prévention et promotion de la santé, Centre intégré universitaire de santé et de services sociaux de la Mauricie et du Centre-du-Québec, Trois-Rivières, Canada; 3 Department of Family Medicine and Emergency Medicine, Faculty of Medicine, Université de Montréal, Montréal, Canada; 4 Present: Department of Nursing, Université du Québec à Trois-Rivières, 3351, boul. des Forges, C.P. 500, Local 4802, Pavillon Santé, Trois-Rivières, QC G8Z 4M3 Canada

**Keywords:** Physical activity, Assessment, Promotion, Healthcare professionals, Primary care, Chronic diseases

## Abstract

**Background:**

Healthcare professionals working in primary care are well positioned to provide initial physical activity counseling owing to their frequent patient contact and their role as a trusted source of health information. Unfortunately, despite health benefits and recommendations, physical activity assessment and promotion in primary care settings is not a routine practice. Because there are currently no standardized practices, it is essential to know what practices are currently employed and explore the barriers to their use. The aim of this study is twofold: first, to describe and compare the practices used by primary care healthcare professionals regarding physical activity assessment and promotion among chronic disease patients and second, to explore the barriers to their use.

**Methods:**

A cross-sectional survey was conducted among primary care healthcare professionals. The questionnaire designed for this project on physical activity assessment and promotion practices was sent electronically to all 619 primary care healthcare professionals in the Mauricie and Centre-du-Quebec regions. Each item was evaluated with a 5-point Likert scale. Descriptive statistics were used to describe sociodemographic characteristics as well as physical activity assessment and promotion practices. Group comparisons were performed according to professionals’ roles.

**Results:**

Sixty-eight (11%) of the healthcare professionals responded to the questionnaire. Both physical activity assessment and promotion practices were reported at similar levels, with mean scores of 3.79 ± 1.05 and 3.69 ± 0.78, respectively, on a 5-point Likert scale. A few specific tools and methods are used by healthcare professionals to assess and promote physical activity. The main barriers to physical activity assessment and promotion during clinical consultation were patients’ perceived lack of interest in physical activity and professionals’ lack of knowledge regarding PA guidelines, tools and methods, and contraindications.

**Conclusions:**

Overall, primary care healthcare professionals are convinced of the benefits of physical activity in preventing and treating chronic diseases and recognize they have an important role to play. Although committed to promoting physical activity among chronic disease patients, they require continuous training on guidelines, validated tools, and motivational interviews together with clear advice to properly implement physical activity assessment and promotion practices.

**Supplementary Information:**

The online version contains supplementary material available at 10.1186/s12875-025-03138-9.

## Background

Chronic diseases (CD) or non-communicable diseases (NCDs) such as cardiovascular diseases, cancers, respiratory diseases, and diabetes were responsible for the deaths of at least 43 million people in 2021 [1]. In Canada, in 2021, 45% of Canadians were living with at least one major chronic disease, and 1 in 12 suffered from three or more chronic conditions [2]. Each year, millions of people are diagnosed with a CD that leads to decreased quality of life and shortened life expectancy [3]. Health-related behaviors, however, provide opportunities for CD prevention [4]. Lifestyle changes, including healthy eating and physical activity (PA), contribute directly to positive health outcomes and can also modify other risk factors [5]. Many studies demonstrate that incorporating daily PA through life routines and increasing cardiorespiratory fitness reduces disease risk [6]. Thus, improving the population-level of PA remains essential for reducing the burden of CDs.

Primary care healthcare professionals (HCPs) are well positioned to provide initial PA counseling owing to their frequent patient contact, their role as a trusted source of health information, and the diversity of HCPs working in primary care clinics [7]. A meta-analysis of 46 randomized controlled trials involving approximately 16,000 participants worldwide reports that PA interventions delivered by primary care HCPs significantly increased moderate to vigorous physical activity (MVPA) in adult patients compared with control groups [8]. A scoping review also reports that encouraging PA in a primary care setting can foster the adoption of PA in individuals with and without a health condition [9]. Systematic review synthesized evidence on the impact of educational interventions aimed at improving physicians’ physical activity counseling and prescription practices, including confidence and knowledge [10]. These findings highlight the importance of supporting healthcare professionals with training and resources to enhance PA promotion in clinical settings.

Indeed, one of the policies in the Global Action Plan on Physical Activity, is to strengthen healthcare systems by increasing the integration of PA assessment, promotion, and prescription within clinical practice [11]. Healthcare systems across the world are being called on to incorporate evidence-based PA assessment and promotion strategies [12]. In line with this, a strategy revealed to have a positive impact on patients is PA assessment and promotion in primary care. Indeed, systematic PA assessment in clinical settings can act as a facilitator for PA counseling, referral, and follow-up to increase the level of PA behavior [13]. However, the current healthcare system lacks standardized, easily usable measures or tools for PA assessment and promotion [14]. This is paradoxical, given that numerous reliable and validated tools are available for use in primary care, including wearable trackers, PA diaries, and questionnaires [15–17]. Wearable trackers, in particular, have demonstrated effectiveness in increasing PA among patients with chronic diseases, yet these tools remain rarely implemented in routine practice.

Unfortunately, PA assessment and promotion in a healthcare setting is not a routine practice [18]. In Quebec, a study reporting a significant increase in PA promotion by primary care physicians during the past decade focused for the most part on patients with chronic conditions or risk factors [19]. However, no results could be found on PA assessment practices used by physicians and other HCPs over the years.

As there are currently no standardized practices for assessing and promoting PA in primary care patients, it is essential to learn the practices currently employed by HCPs and explore the barriers to their use before proposing the tools needed. This study aims first, to describe and compare the PA assessment and promotion practices used by primary care HCPs for CD patients and second, to explore the barriers to PA assessment and promotion among primary care HCPs.

## Methods

### Study design and participants

A cross-sectional study was conducted using a sample of HCPs working in primary care in the Mauricie and Centre-du-Quebec (MCQ) region. Since the primary aim was to describe the practices used by healthcare professionals, the sample size was based on the number of available professionals in the target population, which consisted of 619 healthcare professionals (422 physicians and medical residents and 197 other HCPs, including nurses, social workers, kinesiologists, physiotherapists, respiratory therapists and dietitians) working in one of the 26 family medicine groups (FMGs) or 3 university family medicine groups (U-FMGs) in the MCQ region. We anticipated a response rate of around 10%. Inclusion criteria were as follows. Participants had to: (1) be a health or social services professional practicing in an FMG or U-FMG, and (2) have worked with CD patients for at least six months. Exclusion criteria were not applicable for this project. Potential participants were identified through a convenience sample provided by two main areas of management from the Centre intégré universitaire de santé et de services sociaux de la Mauricie et du Centre-du-Québec (CIUSSS MCQ) and taken from a pool including all primary care HCPs and physicians as of October 2019. To preserve confidentiality and allow for meaningful statistical analysis, professionals categories with very small numbers (e.g., kinesiologists, physiotherapists, respiratory therapists, and dietitians) were categorized as “other HCPs.”

### Study setting

Quebec’s FMGs are organized as private-public partnerships consisting of groups of physicians working closely with other HCPs (i.e., registered nurses and nurse practitioners, social workers, pharmacists, dietitians, physiotherapists, kinesiologists, and psychologists) to provide care for the registered population. Operating costs are shared by the Ministry of Health and Social Services and the physicians. Each FMG and U-FMG is affiliated with the public health and social services public network. The extent of funding for FMGs’ administrative and professional resources is based on the number of patients enrolled. As this number rises and reaches pre-set levels, additional funding is provided to hire more HCPs and administrative staff [20]. FMG objectives are to help ensure the quality and accessibility of primary care and better management of patients’ health.

### Questionnaire

Consistent with a literature review on the PA assessment and promotion practices used by HCPs working with a chronically ill population, a questionnaire was developed specific to this study, with the Consolidated Framework for Implementation Research (CFIR) serving as a reference framework [21]. After the authors (GL, JH) composed the questions, each was reviewed and validated individually by seven experts working in an FMG (i.e., registered nurses, social workers, kinesiologists and general practitioners). To ensure clarity and relevance, semi-structured telephone interviews were conducted with experts representing the target population. To standardize the structure of each interview, we followed a pre-established guide. Experts answered the questionnaire orally and provided feedback on wording, content, and clinical applicability. Minor revisions were made based on their input to improve coherence and alignment with practice.

The final version of the questionnaire comprised a total of 37 questions covering clinical PA assessment and promotion practices and the CFIR dimension related to the implementation of promotion. Apart from two open-ended questions, a 5-point Likert scale was used to capture responses of frequency (1 = never to 5 = always) or agreement (1 = strongly disagree to 5 = strongly agree). Participants were also asked to report sociodemographic characteristics (i.e., gender, age, education level, profession, years of experience, years of experience with chronically ill patients, employment status, and percentage of CD patients followed in their practice). An additional file shows the questionnaire translated into English [see Additional file 1].

### Data collection

The anonymous and confidential questionnaire was sent along with a consent form to potential participants (i.e., physicians, registered nurses, dietitians, kinesiologists, respiratory therapists, physiotherapists, and social workers), via a professional email address, by their program manager or by management between March 2020 and June 2020. It could be completed in about 20 min using the platform of the Université du Québec à Trois-Rivières. Respondents were free to withdraw from the project at any time simply by leaving the platform without saving their answers. HCPs had two weeks to reply to the questionnaire, and a reminder was sent two weeks after the first mailing.

### Ethical approval

The study was conducted in accordance with the Declaration of Helsinki and was fully approved by the Ethics Committee of the CIUSSS MCQ (CERM-2019-008-01).

### Statistical analysis

Descriptive statistics (means, standard deviations, and percentages) were used to describe sociodemographic characteristics and PA assessment and promotion practices. To describe the practices of HCPs, the sample was divided into four groups: nurses, physicians and residents, social workers, and other HCPs (i.e., kinesiologists, physiotherapists, respiratory therapists, and dietitians). The Kruskal-Wallis (H) test was used to compare variables based on different groups of professionals. Homogeneity of variance was verified using the Levene test and showed that the groups were similar in terms of sociodemographic characteristics and work experience. *P*-values < 0.05 were considered statistically significant. All data analyses were performed using version 27.0 of the SPSS Statistics software (IBM Corp., Armonk, NY, USA).

## Results

The questionnaire was completed by 68 participants for a response rate of 11%. Sociodemographic characteristics are presented in Table 1. The majority were women (85.3%), and the mean age of HCPs was 38.0 ± 9 years old. Most respondents were full-time employees (89.7%) and university graduates (94.1%). Their experience working with CD patients varied widely, ranging from 0 to 39 years (*m* = 6.78 *±* 7.21). Among the different groups of HCPs, a statistically significant difference was noted in relation to the number of years of experience working with chronically ill patients (*F* = 3.16; *p* = 0.03). Social workers were the group with the least experience (2 ± 1 years), while physicians reported the most years of experience (9 ± 9 years). Nurses had the highest percentage of CD patients (82.47 ± 16.31%), while social workers had the lowest (25.67 *±* 19.60%).

**Table 1:** Sociodemographic characteristics of participants

Sociodemographic variables	All	Nurses	Physicians	Social workers	Other HCPs
	*n* = 68	*n* = 17	*n* = 31	*n* = 11	*n* = 9
Gender, *n* (%)					
− Men	9 (13.2)	1 (5.9)	5 (16.1)	1 (9.1)	2 (22.2)
− Women	58 (85.3)	16 (94.1)	25 (80.6)	10 (90.9)	7 (77.8)
− Others	1 (1.5)	0 (0)	1 (3.2)	0 (0)	0 (0)
Age, *m* (SD) (*n* = 66)	38 (9)	37 (6)	36 (10)	41 (11)	33 (5)
Education, *n* (%)					
− Technical college	4 (5.9)	2 (11.1)	0 (0)	1 (9.1)	1 (11.1)
− Undergraduate	54 (79.4)	12 (70.6)	26 (83.9)	9 (81.8)	7 (77.8)
− Graduate studies	6 (8.8)	3 (16.7)	1 (3.2)	1 (9.1)	1 (11.1)
− Postgraduate studies	4 (5.9)	0 (0)	4 (12.9)	0 (0)	0 (0)
Years of experience, m (SD)					
− Since graduation (*n* = 67)	11 (9)	13 (6)	10 (10)	15 (8)	8 (6)
− With chronically ill patients	7 (7)	6 (5)	9 (9)	2 (1)	5 (4)
Employment status, *n* (%)					
− Full-time permanent	61 (89.7)	15 (88.2)	31 (100.0)	9 (81.9)	6 (66.7)
− Full-time temporary	2 (2.9)	1 (5.9)	0 (0)	0 (0)	1 (11.1)
− Permanent part-time	4 (5.9)	1 (5.9)	0 (0)	1 (9.1)	2 (22.2)
− Occasional part-time	1 (1.5)	0 (0)	0 (0)	1 (9.1)	0 (0)
Approximate percentage of chronic disease patients followed in their practice	57.9 (26.7)	82.5 (16.3)	50.1(19.3)	25.7 (19.6)	69.9 (24.1)

For most HCPs, beliefs and perceptions of PA were positive. They agreed that PA contributes to health (97%), that sedentary behavior is unhealthy (95%), and that HCPs have a duty to promote PA among their patients (100%). What’s more, respondents were confident in their ability to recommend PA (80%) and stated they intended to recommend it more frequently (90%). Few said they were unable to convince users to be physically active (10.3%).

Table 2 depicts the frequency of PA assessment and promotion practices used by primary HCPs. Both PA assessment and promotion practices were used at a similar frequency, which is 3.79 ± 1.05 and 3.69 ± 0.78, respectively, on a 5-point Likert scale. However, a comparison between groups revealed that nurses and other HCPs used assessment more often, although this was less true for social workers (*p* < 0.01). PA promotion was used at a similar frequency for each group. No statistically significant differences between groups were detected, suggesting similar assessment and promotion practices for each group of stakeholders.

**Table 2:** Frequency of PA assessment and intervention practices

	All *n* = 68	Nurses *n* = 17	Physicians *n* = 31	Social workers (*n* = 11)	Other HCPs *n* = 9
Frequency of PA assessment, m (SD)	3.79 (1.05)	4.47 (0.51)	3.58 (0.77)	2.82 (1.54)	4.44 (0.73)
Frequency of PA intervention, m (SD)	3.69 (0.78)	3.71 (0.69)	3.68 (0.75)	3.55 (1.04)	3.89 (0.78)

1 = never, 2 = rarely, 3 = sometimes, 4 = often, 5 = always

**Fig. 1:**
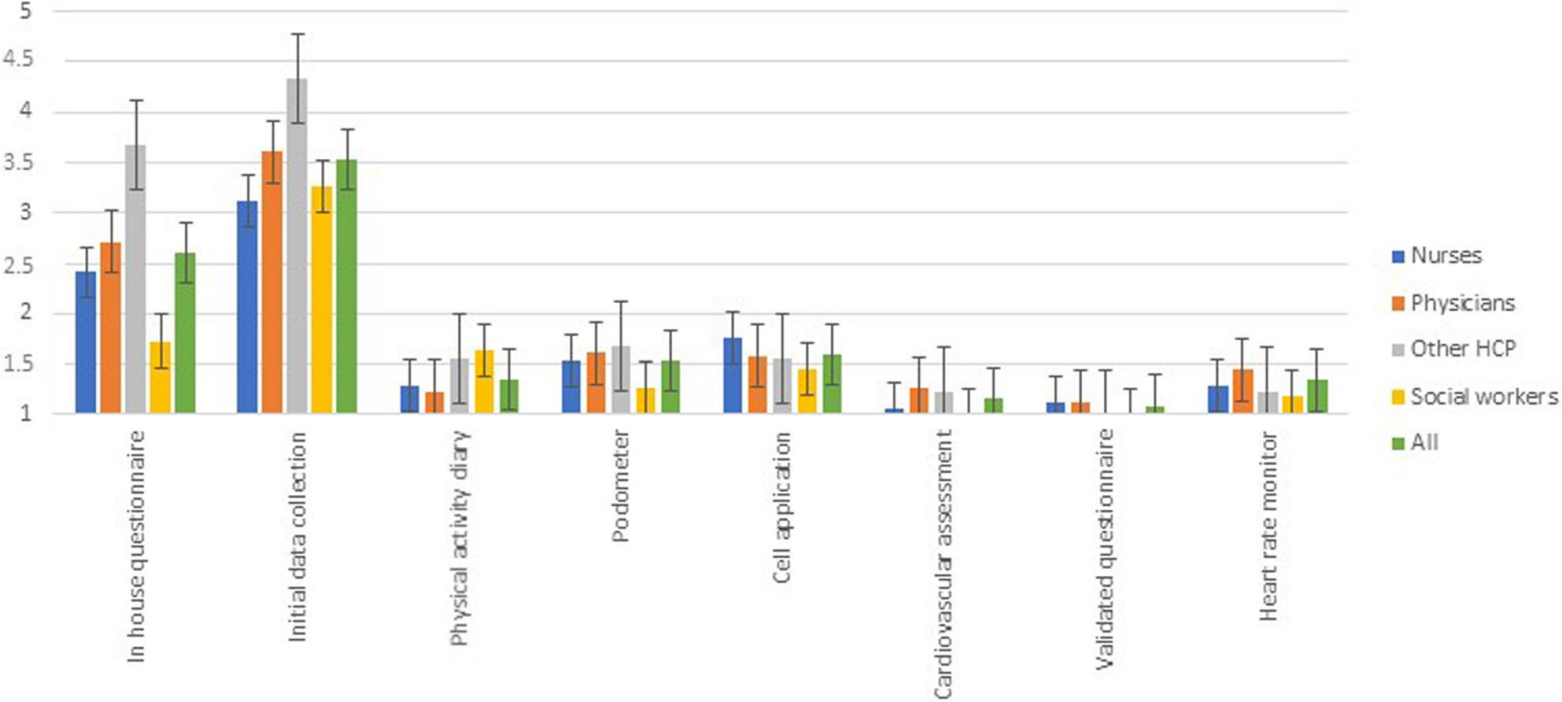
Frequency of use of PA assessment tools. Legend: 1=never, 2=rarely, 3=sometimes, 4=often, 5= always

The survey also identified the key topics discussed during the PA assessment and promotion segment of the clinical consultation. Results, for the most part, show that leisure-time PA (4.20 ± 0.86) and type of PA (4.37 ± 0.75) practiced are the items most often discussed during consultations, whereas sedentary behavior receives less attention (2.53 ± 1.13). All HCP groups discuss barriers to PA (3.88 ± 0.89) more often than factors facilitating PA practice (3.06 ± 1.08) and employ very few specific tools and methods to assess and promote PA. Figure 1 demonstrates the frequency of use of PA assessment tools. HCPs tend to use the life habit section of a data collection form found in clinics (3.53 ± 1.62); otherwise, they never, rarely, or sometimes use wearable trackers (such as pedometers or accelerometers), PA diaries, validated questionnaires to assess PA, or mobile applications. Like other HCP groups that promote PA, HCPs mainly favor leaflets or written prescriptions (Fig 1).

Table 3 presents barriers to routine PA assessment and promotion in primary care practice. The perception of patients’ lack of interest in PA is the most frequent barrier reported by all HCPs (4.01 ± 0.95), except social workers (3.27 ± 1.19). Physicians report that lack of time is almost always a barrier to PA assessment (4.74 ± 0.63) or promotion (4.87 ± 0.34), but is not caused by a lack of knowledge. Nurses and social workers, conversely, report that lack of knowledge is a major barrier to PA assessment and promotion, with specific regard to the related guidelines, tools and methods, and contraindications. These two groups also report that their lack of knowledge pertaining to PA data interpretation and contraindications are barriers to PA assessment and promotion with their patients. Other barriers are mentioned but are less significant than perceived patient disinterest and lack of time.

**Table 3:** Barriers to PA assessment and promotion in primary care setting

	All *n* = 68	Nurses *n* = 17	Physicians *n* = 31	Social workers *n* = 11	Other HCPs *n* = 9
Perception of patients’ lack of interest in physical activity	4.01 (0.95)	4.35 (0.49)	4.03 (0.84)	3.27 (1.19)	4.22 (1.30)
Lack of time for PA assessment	3.87 (1.33)	3.53 (1.42)	4.74 (0.63)	2.55 (1.13)	3.11 (1.17)
Lack of time for PA promotion	3.97 (1.29)	3.47 (1.42)	4.87 (0.34)	2.64 (1.12)	3.44 (1.24)
Lack of knowledge about PA assessment tools	3.91 (1.17)	4.47 (0.62)	3.55 (1.23)	4.27 (0.91)	3.67(1.58)
Lack of knowledge about PA promotion methods	3.91 (1.03)	4.47 (0.51)	3.55 (0.96)	4.55 (0.52)	3.33 (1.58)
Lack of knowledge about PA data interpretation	3.63 (1.35)	4.41 (0.71)	2.94 (1.26)	4.64 (0.51)	3.33 (1.80)
Lack of knowledge about PA contraindications	3.21 (1.28)	4.06 (0.90)	2.39 (0.88)	4.27 (0.65)	3.11 (1.69)
Lack of knowledge about PA promotion guidelines	3.12 (1.15)	3.88 (0.78)	2.55 (0.81)	4.09 (1.04)	2.44 (1.33)
Lack of knowledge about PA assessment guidelines	2.91 (1.21)	3.53 (0.87)	2.35 (0.95)	3.91 (1.22)	2.44 (1.33)

Legend: 1 = never, 2 = rarely, 3 = sometimes, 4 = often, 5 = always

## Discussion

This paper demonstrates the frequency of use of physical activity assessment and promotion in primary care for adults with chronic diseases. Results show that HCPs working in primary care understand the benefits of PA, a fact reflected in their fairly frequent self-reported use of PA assessment and promotion. This is encouraging, as positive attitudes and beliefs are key facilitators to implementing community-based PA interventions [22]. A systematic review also demonstrates that between 42.5% and 61% of HCPs (i.e., general practitioners, physicians, nurses, physiotherapists, exercise physiologists, dietitians, diabetes health educators, pharmacists, surgeons, podiatrists, oncologists, occupational and physical therapists, and healthcare assistants) surveyed, use PA assessment, with the majority promoting PA through some form of related advice and/or counseling [23]. Consistent with our results, they also agree that PA promotion is part of their role [23].

Our study reveals various degrees of frequency of PA assessment among different groups of HCPs. For example, nurses and the other HCP groups (i.e., kinesiologists, physiotherapists, respiratory therapists, and dietitians) reported performing more PA assessments than social workers. Nurses and social workers, however, also said that a lack of knowledge about PA assessment tools is a frequent barrier to assessing this behavior. To facilitate implementation of PA promotion, we recommend placing greater emphasis on HCPs’ individual skills and active involvement, as both are essential to enhance self-efficacy and knowledge [22].

Frequency of PA promotion was similar for each HCP group, but social workers and nurses reported a greater number of barriers. Social workers are in a unique position to promote enjoyable PA because they can help patients negotiate barriers and use personal strengths. They are also aware of the community resources that can assist them [24]. Nurses interacting closely and frequently with patients are also well placed to promote PA [25]. However, since it has been shown that PA assessment and promotion go hand in hand [26] and that assessments guide interventions [23, 27–30], both social workers and nurses need clear advice, increased education, and greater knowledge of the guidelines [25].

As part of the consultation, the HCPs surveyed reported they tend not to discuss sedentary time or time spent in sedentary behaviors with their patients, preferring to talk more about type of PA practiced or leisure-time PA. A study also evaluated sedentary behavior counseling practices in primary care, which, as our results demonstrate, appear infrequent [31]. And yet, high volumes of sedentary behavior are associated with all-cause mortality and the development of CD, including cardiovascular disease, type 2 diabetes, and some cancers [32]. Indeed, an intervention aimed at progress toward active living may preferably be preceded by a decrease in sedentary time [33]. Measuring sedentary behavior is therefore important, especially in less active patient populations where treatment-related improvements are first noticed following a change in sedentary behavior as opposed to PA measures [34]. While devices such as accelerometers and inclinometers are increasingly used for assessing these behaviors, they provide limited contextual information to explain where and how individuals are sedentary [35]. Multi-item questionnaires respect accuracy but present a high degree of variability [35]. Whenever possible, therefore, HCPs should consider using both methods to capture total time spent in sedentary behavior, considering the method’s appropriateness and relevance for the population of interest [36].

As part of this study, HCPs also identified barriers to use of routine PA assessment and promotion in practice. Although patients’ perceived lack of interest is reportedly a frequent barrier to PA assessment and promotion, a meta-analysis reveals that HCPs’ use of motivational interviews may have a small positive effect on self-reported physical activity in those with chronic health conditions [37]. Given sufficient training, most HCPs could incorporate motivational interviewing into clinical practice and thereby increase patients’ interest in physical activity [37]. Physicians also mention lack of time during consultations, while nurses and social workers mention lack of knowledge. These results align with a systematic review of 17 studies on HCPs indicating that limited counseling time is a barrier to PA promotion. As well, 9 of the 17 studies reported that lack of PA-related knowledge or training is a further barrier to assessment and promotion [23]. Given these barriers, all clinical staff members must be trained regarding PA, to properly use assessment tools [38], adhere to promotion guidelines, and know the contraindications.

Although they report that PA assessment and promotion is relatively frequent in their practice, primary care HCPs seldom use validated tools (i.e., pedometer, heart rate monitor, validated questionnaire, PA diary, etc.). The tool most in favor is a data collection form that includes questions on type of PA practiced and its weekly frequency; this type of form, however, is less accurate and often contains overestimations of PA. This suggests that many HCPs assess PA informally, through general questions or patient self-report, rather than using validated instruments. As a vital sign, PA level can be tracked over time, facilitating more comprehensive and personalized counseling initiatives [38]. Several validated PA assessment tools such as questionnaires and monitors are available [28, 29, 39]. With respect to questionnaires, the General Practice Physical Activity Questionnaire (GPPAQ) is the one most frequently mentioned in the literature to assess physical activity in primary care [28]. Several studies have already revealed the effectiveness of pedometers and accelerometers to assess PA levels and intervene with CD patients [40, 41]. A systematic review and a meta-analysis also indicate that the combined use of a monitoring device (particularly a pedometer) and regular consultations with HCPs leads to the greatest PA improvements in individuals with cardiometabolic conditions [42]. As well, an umbrella review on the effectiveness of a wearable activity-tracker-based intervention provides consistent evidence that wearable activity trackers effectively increase PA in a wide variety of clinical and non-clinical populations and age groups [43]. The increasing use of consumer wearable devices to regularly monitor PA could offer an opportunity to assimilate data collected from devices into routine health monitoring, leading to more tailored activity prescriptions and improved patient health profiles and outcomes [38].

### Strengths and limitations

The main strength of this study is that it involved all members of the multidisciplinary team working in primary care, including social workers. Thanks to their position and knowledge, social workers and other psychosocial workers as well are capable of influencing changes in patient behavior. Thus, it’s important to learn their interests and knowledge of PA to enable them to better support PA promotion. Because a limitation of this study is the relatively small number of professionals surveyed, it may be impossible to generalize its findings to all HCPs in the province of Quebec. Additionally, certain professions with very few participants (e.g., kinesiologists, physiotherapists, respiratory therapists, and dietitians) were grouped under “other HCPs” to preserve confidentiality and allow for statistical analysis. This grouping may have reduced the specificity of profession-related differences in roles and practices. Another limitation is that the reported barriers to PA assessment and promotion were captured only through predefined questionnaire items, although an open-text field for additional input was available. A more in-depth exploration using qualitative methods could have provided richer insights into the underlying causes of these barriers, such as knowledge gaps or time constraints [44]. The results are based solely on frequency of use and not on content pertaining to evaluation or promotion. Participation in this study was voluntary. Selection bias must also be considered, since participants who took the time to participate were likely interested in PA, whereas non-respondents were not. Furthermore, data were collected during the COVID-19 pandemic, which may have influenced the response rate and the answers given. Another bias concerns social desirability. Respondents may have been inclined to give answers deemed desirable, possibly leading to overestimation of the frequency of PA assessment and promotion.

## Conclusions

Despite its limitations, this descriptive study reveals the frequency of current practices in PA assessment and promotion among HCPs working in FMGs and U-FMGs in the Maurice and Centre-du-Québec sanitary region of Quebec. It’s encouraging to see that HCPs understand the benefits of PA in preventing and treating CDs and recognize they have a role to play in its promotion. Although those practicing in FMGs are committed to promoting PA among CD patients, few validated, evidence-based tools are currently used to assess or promote it. As a result, HCPs working with CD patients need ongoing training on validated assessment and promotion tools and innovative devices that can be incorporated simply and congruently into regular clinical routines, as this may help enhance their patients’ individual PA levels and reduce sedentary behavior.

## Supplementary Information

Supplementary Material 1.

## Data Availability

Data used to support this study’s findings are available from the corresponding author upon request.

## Abbreviations

CD: chronic disease
CFIR: Consolidated Framework for Implementation Research
CISSS: Integrated health and social services centres
CIUSSS: Integrated university health and social services centres
CIUSSS MCQ: Centre Intégré universitaire de santé et de services sociaux de la Mauricie et du Centre-du-Québec
FMG: Family medicine group
GPPAQ: General Practice Physical Activity Questionnaire
HCPs: Healthcare professionals
MCQ: Mauricie and Centre-du-Quebec
MSSS: Ministry of Health and Social Services
NCDs: Non-communicable diseases
PA: physical activity
U-FMG: University family medicine group
WHO: World Health Organization

## References

[1] World Health Organization. Non-communicable diseases. 2014. https://www.who.int/news-room/fact-sheets/detail/noncommunicable-diseases. Accessed 3 Feb 2025.

[2] Statistics Canada. A glimpse at the health of Canadians. 2023. https://www.statcan.gc.ca/o1/en/plus/5102-glimpse-health-canadians. Accessed 3 Feb 03 2025.

[3] Rattay KT, Henry LMG, Killingsworth RE. Preventing chronic disease: the vision of public health. Dela J Public Health. 2017;3(2):52–6. 34466910 10.32481/djph.2017.04.008 PMC8352511

[4] Rahelić V, Perković T, Romić L, Perković P, Klobučar S, Pavić E, et al. The role of behavioral factors on chronic diseases-practice and knowledge gaps. Healthc (Basel). 2024;12(24):2520. 10.3390/healthcare12242520 PMC11675894 39765947

[5] Koehler K, Drenowatz C. Integrated role of nutrition and physical activity for lifelong health. Nutrients. 2019;11(7):1437. 31247924 10.3390/nu11071437 PMC6682932

[6] Anderson E, Durstine JL. Physical activity, exercise, and chronic diseases: A brief review. Sports Med Health Sci. 2019;1(1):3–10. 35782456 10.1016/j.smhs.2019.08.006 PMC9219321

[7] Jones M, Bright P, Hansen L, Ihnatsenka O, Carek PJ. Promoting physical activity in a primary care practice: overcoming the barriers. Am J Lifestyle Med. 2019;15(2):158–64. 33786031 10.1177/1559827619867693 PMC7958222

[8] Kettle VE, Madigan CD, Coombe A, Graham H, Thomas JJC, Chalkley AE, et al. Effectiveness of physical activity interventions delivered or prompted by health professionals in primary care settings: systematic review and meta-analysis of randomised controlled trials. BMJ. 2022;376:e068465. 35197242 10.1136/bmj-2021-068465 PMC8864760

[9] Grogg KA, Giacobbi PR, Blair EK, Haggerty TS, Lilly CL, Winters CS, et al. Physical activity assessment and promotion in clinical settings in the united states: a scoping review. Am J Health Promotion. 2022;36(4):714–37. 10.1177/08901171211051840 35224998

[10] Courish MK, Shivgulam ME, Petterson JL, Pellerine L, Kivell MJ, Wilson T, Theou O, O’Brien MW. A review of educational interventions on physicians’ exercise counseling and prescription practices. Transl Am Coll Sports Med. 2024;9(1):1–7.

[11] World Health Organization. Global action plan on physical activity 2018–2030: more active people for a healthier world. Geneva: World Health Organization; 2019.

[12] Lin JS, O’Connor E, Evans CV, Senger CA, Rowland MG, Groom HC. Behavioral counseling to promote a healthy lifestyle in persons with cardiovascular risk factors: a systematic review for the U.S. Preventive services task force. Ann Intern Med. 2014;161:568–78. 25155549 10.7326/M14-0130

[13] Arena R, Guazzi M, Lianov L, Whitsel L, Berra K, Lavie CJ et al. Healthy lifestyle interventions to combat noncommunicable disease—a novel nonhierarchical connectivity model for key stakeholders: a policy statement from the American Heart Association, European Society of Cardiology, European Association for Cardiovascular Prevention and Rehabilitation, and the American College of Preventive Medicine. Mayo Clin Proc. 2015; 90(8):1082–1103. 10.1016/j.mayocp.2015.05.001 26143646

[14] Whitsel LP, Bantham A, Jarrin R, Sanders L, Stoutenberg M. Physical activity assessment, prescription and referral in US healthcare: how do we make this a standard of clinical practice? Prog Cardiovasc Dis. 2021;64:88–95. 33383058 10.1016/j.pcad.2020.12.006

[15] Franssen WMA, Franssen GHLM, Spaas J, Solmi F, Eijnde BO. Can consumer wearable activity tracker-based interventions improve physical activity and cardiometabolic health in patients with chronic diseases? A systematic review and meta-analysis of randomised controlled trials. Int J Behav Nutr Phys Act. 2020;17(1):57. 32393357 10.1186/s12966-020-00955-2 PMC7216601

[16] Hodkinson A, Kontopantelis E, Zghebi SS, Grigoroglou C, McMillan B, Marwijk HV, et al. Association between patient factors and the effectiveness of wearable trackers at increasing the number of steps per day among adults with cardiometabolic conditions: meta-analysis of individual patient data from randomized controlled trials. J Med Internet Res. 2022;24(8):e36337. 36040779 10.2196/36337 PMC9472038

[17] Smith TO, McKenna MC, Salter C, Hardeman W, Richardson K. Hillsdon Met al. A systematic review of the physical activity assessment tools used in primary care. Fam Pract. 2017;34(4):384–91. 28334801 10.1093/fampra/cmx011

[18] Whitsel LP, Bantham A, Chase PJ, Dunn P, Hovind L, McSwain B. The current state of physical activity assessment and interventions with public policy solutions. Prog Cardiovasc Dis. 2024;83:29–35. 38428786 10.1016/j.pcad.2024.02.012

[19] Laberge S, Gosselin V, Lestage K, Chagnon M, Guimond C. Promotion of physical activity by Québec primary care physicians: what has changed in the last decade? J Phys Act Health. 2024;21(5):508–18. 38490193 10.1123/jpah.2023-0379

[20] Ministère de la Santé et des Services sociaux (MSSS). Integration Sheets for Professionals in GMF. Department of Health and Social Services Publications. 2017. https://publications.msss.gouv.qc.ca/msss/document-001529/?&txt=guides%27int%C3%A9gration&msss_valpub&date=DESC. Accessed 19 July 2022.

[21] Damschroder LJ, Reardon CM, Widerquist MAO, Lowery J. The updated consolidated framework for implementation research based on user feedback. Implement Sci. 2022;17(1):75. 36309746 10.1186/s13012-022-01245-0 PMC9617234

[22] Cooper J, Murphy J, Woods C, Van Nassau F, McGrath A, Callaghan D, et al. Barriers and facilitators to implementing community-based physical activity interventions: a qualitative systematic review. Int J Behav Nutr Phys Act. 2021;18:118. 34493306 10.1186/s12966-021-01177-w PMC8422651

[23] Albert FA, Crowe MJ, Malau-Aduli AEO, Malau-Aduli BS. Physical activity promotion: A systematic review of the perceptions of healthcare professionals. Int J Environ Res Public Health. 2020;17(12):4358. 32570715 10.3390/ijerph17124358 PMC7345303

[24] Williams DJ, Strean WB. Physical activity promotion in social work. Soc Work. 2006;51(2):180–4. 16858925 10.1093/sw/51.2.180

[25] van Hell-Cromwijk M, Metzelthin SF, Schoonhoven L, Verstraten C, Kroeze W, de Man van Ginkel JM. Nurses’ perceptions of their role with respect to promoting physical activity in adult patients: a systematic review. J Clin Nurs. 2021;30(17–18):2540–62. 33899286 10.1111/jocn.15747

[26] Crisford P, Winzenberg T, Venn A, Schultz M, Aitken D, Cleland V. Factors associated with physical activity promotion by allied and other nonmedical health professionals: A systematic review. Patient Educ Couns. 2018;101(10):1775–85. 29793786 10.1016/j.pec.2018.05.011

[27] Pettee Gabriel K, Morrow JR, Woolsey AL. Framework for physical activity as a complex and multidimensional behaviour. J Phys Act Health. 2012;9(Suppl 1):11–8. 10.1123/jpah.9.s1.s11 22287443

[28] Smith TO, McKenna MC, Salter C, Hardeman W, Richardson K, Hillsdon M, et al. A systematic review of the physical activity assessment tools used in primary care. Family Practic. 2017;34(4):384–91. 10.1093/fampra/cmx011 28334801

[29] Strath SJ, Kaminsky LA, Ainsworth BE, Ekelund U, Freedson PS, Gary RA, et al. Guide to the assessment of physical activity: clinical and research applications: a scientific statement from the American heart association. Circulation. 2013;128(20):2259–79. 24126387 10.1161/01.cir.0000435708.67487.da

[30] Wald A, Garber CE. A review of current literature on vital sign assessment of physical activity in primary care. J Nurs Scholarsh. 2018;50(1):65–73. 29068556 10.1111/jnu.12351

[31] Shuval K, DiPietro L, Skinner CS, Barlow CE, Morrow J, Goldsteen R, et al. Sedentary behaviour counselling: the next step in lifestyle counselling in primary care; pilot findings from the rapid assessment disuse index (RADI) study. Br J Sports Med. 2014;48(19):1451–5. 22976910 10.1136/bjsports-2012-091357 PMC4229046

[32] Dempsey PC, Matthews CE, Dashti SG, Doherty AR, Bergouignan A, van Roekel EH, et al. Sedentary behavior and chronic disease: mechanisms and future directions. J Phys Act Health. 2020;17(1):52–61. 31794961 10.1123/jpah.2019-0377

[33] Dogra S, Copeland JL, Altenburg TM, Heyland DK, Owen N, Dunstan DW. Start with reducing sedentary behavior: a Stepwise approach to physical activity counseling in clinical practice. Patient Educ Couns. 2022;105(6):1353–61. 34556383 10.1016/j.pec.2021.09.019

[34] Byrom B, Stratton G, Mc Carthy M, Muehlhausen W. Objective measurement of sedentary behaviour using accelerometers. Int J Obes (Lond). 2016;40(11):1809–12. 27478922 10.1038/ijo.2016.136 PMC5116050

[35] Prince SA, Cardilli L, Reed JL, Saunders TJ, Kite C, Douillette K, et al. A comparison of self-reported and device measured sedentary behaviour in adults: a systematic review and meta-analysis. Int J Behav Nutr Phys Act. 2020;17(1):31. 32131845 10.1186/s12966-020-00938-3 PMC7055033

[36] Aunger J, Wagnild J. Objective and subjective measurement of sedentary behavior in human adults: A toolkit. Am J Hum Biol. 2022;34(1):e23546. 33277954 10.1002/ajhb.23546 PMC9286366

[37] O’Halloran PD, Blackstock F, Shields N, Holland A, Iles R, Kingsley M, et al. Motivational interviewing to increase physical activity in people with chronic health conditions: a systematic review and meta-analysis. Clin Rehabil. 2014;28(12):1159–71. 24942478 10.1177/0269215514536210

[38] Stoutenberg M, Shaya GE, Feldman DI, Carroll JK. Practical strategies for assessing patient physical activity levels in primary care. Mayo Clin Proc Innov Qual Outcomes. 2017;1(1):8–15. 30225397 10.1016/j.mayocpiqo.2017.04.006 PMC6134906

[39] Lobelo F, Rohm Young D, Sallis R, Garber MD, Billinger SA, Duperly J, et al. Routine assessment and promotion of physical activity in healthcare settings: A scientific statement from the American heart association. Circulation. 2018;137(18):e495–522. 29618598 10.1161/CIR.0000000000000559

[40] Dasgupta K, Rosenberg E, Joseph L, Cooke AB, Trudeau L, Bacon SL, et al. Physician step prescription and monitoring to improve arterial health (SMARTER): A randomized controlled trial in patients with type 2 diabetes and hypertension. Diabetes Obes Metab. 2017;19(5):695–704. 28074635 10.1111/dom.12874 PMC5412851

[41] Houle J, Doyon O, Vadeboncoeur N, Turbide G, Diaz A, Poirier P. Effectiveness of a pedometer-based program using a socio-cognitive intervention on physical activity and quality of life in a setting of cardiac rehabilitation. Can J Cardiol. 2012;28:27–32. 22177854 10.1016/j.cjca.2011.09.020

[42] Hodkinson A, Kontopantelis E, Adeniji C, van Marwijk H, McMillian B, Bower P, et al. Interventions using wearable physical activity trackers among adults with cardiometabolic conditions: A systematic review and meta-analysis. JAMA Netw Open. 2021;4(7):e2116382. 34283229 10.1001/jamanetworkopen.2021.16382 PMC9387744

[43] Ferguson T, Olds T, Curtis R, Blake H, Crozier AJ, Dankiw K, et al. Effectiveness of wearable activity trackers to increase physical activity and improve health: a systematic review of systematic reviews and meta-analyses. Lancet Digit Health. 2022;4(8):e615–26. 35868813 10.1016/S2589-7500(22)00111-X

[44] Johnson RB, Onwuegbuzie AJ, Turner LA. Toward a definition of mixed methods research. J Mixed Methods Res. 2007;1(2):112–33.

