# Supplementary material for: Healthcare professionals’ practices and barriers in assessing and promoting physical activity in primary care: a descriptive study
Source: BMC Prim Care. 2025 Dec 22;26:407. doi: 10.1186/s12875-025-03138-9 (PMC12752442; doi:10.1186/s12875-025-03138-9)
Supplement: Supplementary file 1 — Supplementary Material 1. [file 12875_2025_3138_MOESM1_ESM.docx]

Questionnaire

Physical activity to prevent chronic disease:
Portrait of interventions in family medicine groups in Mauricie and Centre-du-Quebec regions

**Research objectives**

1. To describe the physical activity assessment and intervention methods used by members of the multidisciplinary team with people with chronic diseases followed in the family medicine group (FMG).

2. To describe the external and internal practice contexts and individual characteristics of these professionals.

Instructions: Answer each question to the best of your knowledge, thinking of the situation representing the last month. If you work in several FMGs or at multiple service points, consider the situation that best represents your general reality. You can return to the question at any time. The estimated time to complete the questionnaire is approximately 20 minutes.

Please remember: the questions are not intended to assess your skills or knowledge, but rather to help us better understand your situation.

**Definitions:**

Chronic diseases: long-term conditions with lifestyle as a risk or protective factor. For example, diabetes, cardiovascular diseases including hypertension, coronary heart disease and heart failure, chronic obstructive pulmonary disease, cancers and depression (WHO, 2018).

Physical activity: complex, multidimensional behavior that increases energy expenditure, whether during leisure, work, travel. or daily tasks (Pettee, 2012).

Thank you for your valuable collaboration!

The research team

**Section 1: Three themes related to professional practices**

| 1. Evaluating physical activity for people with chronic diseases |
| --- |

To answer the following questions, please reflect on the clinical situations you encountered over the past month in relation to your follow-up of individuals living with one or more chronic diseases.

**1. In the FMG, I assess physical activity levels in individuals with chronic diseases?**

Always

Often

Sometimes

Rarely

Never

**2. I evaluate the physical activity of people with chronic diseases:** [For each statement, check off what represents your practice].

|  | Always | Often | Sometimes | Rarely | Never |
| --- | --- | --- | --- | --- | --- |
| At every appointment |  |  |  |  |  |
| For each new user |  |  |  |  |  |
| At a user's request |  |  |  |  |  |
| At the request of another professional |  |  |  |  |  |
| Other (specify) : |  |  |  |  |  |

**3. When assessing the physical activity of people with chronic diseases, do I address the following elements?** [For each statement, check off what represents your practice].

|  | Always | Often | Sometimes | Rarely | Never |
| --- | --- | --- | --- | --- | --- |
| Physical activity for leisure |  |  |  |  |  |
| Physical activity for travel purpose |  |  |  |  |  |
| Physical activity in work or main occupation |  |  |  |  |  |
| Physical activity for daily tasks |  |  |  |  |  |
| The number of minutes of physical activity per week spent at moderate to high intensity. |  |  |  |  |  |
| Number of days per week of moderate-to-high-intensity physical activity lasting at least 10 consecutive minutes. |  |  |  |  |  |
| Duration of each physical activity session |  |  |  |  |  |
| Intensity of physical activity sessions |  |  |  |  |  |
| Types of physical activity (e.g. walking, swimming, yoga, cycling, etc.) |  |  |  |  |  |
| Sedentary activity time (e.g. television, smartphone, video games, etc.) |  |  |  |  |  |
| Motivation to take part in physical actvity |  |  |  |  |  |
| Barriers and limitations (e.g. knee pain, back pain, costs, lack of time, etc.) that prevent you from being physically active |  |  |  |  |  |
| Facilitating factors for physical activity (e.g. accessibility, low cost, time, self-efficacy, etc.). |  |  |  |  |  |
| Other (specify) : |  |  |  |  |  |

**4. When evaluating physical activity, I use the following tools or methods:**
[For each statement, check which represents your practice]

|  | Always | Often | Sometimes | Rarely | Never |
| --- | --- | --- | --- | --- | --- |
| An « in-house » brief questionnaire |  |  |  |  |  |
| The Lifestyle section of the initial data collection |  |  |  |  |  |
| A scientifically validated physical activity evaluation questionnaire (e.g.: General Physical Activity Practice Questionnaire (GPAP), Godin questionnaire, etc.).  Specify : _________________________ |  |  |  |  |  |
| A physical activity diary |  |  |  |  |  |
| A pedometer/accelerometer-type physical activity monitor (e.g. Fitbit) |  |  |  |  |  |
| Heart rate monitor (e.g. polar watch) |  |  |  |  |  |
| An application for smartphones |  |  |  |  |  |
| A submaximal cardiorespiratory capacity test (e.g.: walking, cycling, stair tests) |  |  |  |  |  |
| Other (specify) : |  |  |  |  |  |

**5. The results of the physical activity evaluation are recorded in the patient’s medical record:**

Always

Often

Sometimes

Rarely

Never

**6**. **The evaluation of physical activity for people with chronic diseases allows me to** _____________? [For each statement, check what represents your opinion]

|  | Strongly agree | Agree | Neither agree nor disagree | Disagree | Never |
| --- | --- | --- | --- | --- | --- |
| Check if user is meeting physical activity recommendations |  |  |  |  |  |
| Measure daily energy expenditure |  |  |  |  |  |
| Classify physical activity level (ex: Very active, active, not very active, sedentary) |  |  |  |  |  |
| Guide physical activity intervention |  |  |  |  |  |
| Target a physical activity goal (e.g. 8,000 steps a day, 30-minute walk a day) |  |  |  |  |  |
| Set a goal for achieving a clinical target (e.g. lower blood pressure, reduce anxiety) |  |  |  |  |  |
| Other (specify) :  ________________________________  ________________________________ |  |  |  |  |  |

7. **Physical activity assessments should be carried out by :** ________________
 [For each statement, check the one that represents your opinion]

|  | Strongly agree | Agree | Neither agree nor disagree | Disagree | Never |
| --- | --- | --- | --- | --- | --- |
| Physician |  |  |  |  |  |
| Nurse |  |  |  |  |  |
| Primary care nurse practitioner (PCNP) |  |  |  |  |  |
| Kinesiologist |  |  |  |  |  |
| Social worker |  |  |  |  |  |
| Psychologist |  |  |  |  |  |
| Occupational therapist |  |  |  |  |  |
| Physiotherapist |  |  |  |  |  |
| Professional collecting initial data |  |  |  |  |  |
| Other (specify) :  ______________________________  ______________________________ |  |  |  |  |  |

8. **What are my limitations in assessing physical activity in people with chronic diseases?** [For each statement, check what represents your opinion]

|  | Strongly agree | Agree | Neither agree nor disagree | Disagree | Never |
| --- | --- | --- | --- | --- | --- |
| Lack of time |  |  |  |  |  |
| Lack of knowledge of assessment tools and methods |  |  |  |  |  |
| Lack of knowledge of how to interpret results |  |  |  |  |  |
| Lack of knowledge of physical activity guidelines or good practice |  |  |  |  |  |
| Lack of knowledge about contraindications to physical activity |  |  |  |  |  |
| Lack of interest in physical activity on the part of users |  |  |  |  |  |
| Fear of harming users |  |  |  |  |  |
| Judgment of colleagues |  |  |  |  |  |
| Other (specify) :  ______________________________  ______________________________ |  |  |  |  |  |

9. **What would help me to carry out the physical activity assessment in the best possible way?** [For each statement, check the one that represents your opinion]

|  | Strongly agree | Agree | Neither agree nor disagree | Disagree | Never |
| --- | --- | --- | --- | --- | --- |
| Training |  |  |  |  |  |
| Sharing of existing assessment tools and methods |  |  |  |  |  |
| Access to clinical physical activity assessment tools (e.g. questionnaires, physical activity monitors) |  |  |  |  |  |
| Financial resources to purchase equipment |  |  |  |  |  |
| Access to clinical support (clinical expertise) |  |  |  |  |  |
| Other (specify) :  ______________________________ |  |  |  |  |  |

| **Physical activity intervention for people with chronic diseases** |
| --- |

**10. I carry out physical activity interventions for people with chronic diseases**

Always

Often

Sometimes

Rarely

Never

**11. Physical activity intervention should be carried out by:**_______________

[For each statement, check the one that represents your opinion]

|  | Strongly agree | Agree | Neither agree nor disagree | Disagree | Never |
| --- | --- | --- | --- | --- | --- |
| Physician |  |  |  |  |  |
| Nurse |  |  |  |  |  |
| Primary care nurse practitioner (PCNP) |  |  |  |  |  |
| Kinesiologist |  |  |  |  |  |
| Social worker |  |  |  |  |  |
| Psychologist |  |  |  |  |  |
| Occupational therapist |  |  |  |  |  |
| Physiotherapist |  |  |  |  |  |
| Professional collecting initial data |  |  |  |  |  |
| Other (specify) :  ______________________________  ______________________________ |  |  |  |  |  |

**12. My physical activity interventions are carried out:**______________________

[For each statement, check which represents your practice]

| Following the assessment | Always | Often | Sometimes | Rarely | Never |
| --- | --- | --- | --- | --- | --- |
| When the need/desire is expressed by the person |  |  |  |  |  |
| During follow-up appointments |  |  |  |  |  |
| When a new patient visits |  |  |  |  |  |
| At the request of another professional |  |  |  |  |  |
| Other (specify) :  ______________________________  ______________________________ |  |  |  |  |  |

**13. My physical activity interventions are guided by**:____________________

[For each statement, check which represents your practice]

|  | Always | Often | Sometimes | Rarely | Never |
| --- | --- | --- | --- | --- | --- |
| User motivations |  |  |  |  |  |
| User needs |  |  |  |  |  |
| My intuition |  |  |  |  |  |
| Assessment results |  |  |  |  |  |
| Physical activity recommendations or guidelines |  |  |  |  |  |
| Other (specify) :  ______________________________ |  |  |  |  |  |

**14. When I address physical activity into my interventions, I use the following approach(es):** [For each statement, check what represents your practice]

|  | Always | Often | Sometimes | Rarely | Never |
| --- | --- | --- | --- | --- | --- |
| Motivational interviewing |  |  |  |  |  |
| Education of the individual and his or her family, where appropriate |  |  |  |  |  |
| Group meetings |  |  |  |  |  |
| Other (specify) :  ______________________________ |  |  |  |  |  |

**15. When I incorporate physical activity in my practice, I use the following tool(s):** [For each statement, check what represents your practice]

|  | Always | Often | Sometimes | Rarely | Never |
| --- | --- | --- | --- | --- | --- |
| Physical activity diary |  |  |  |  |  |
| Physical activity prescription |  |  |  |  |  |
| Pedometer/accelerometer-type physical activity monitor |  |  |  |  |  |
| Heart rate monitor (e.g. polar watch) |  |  |  |  |  |
| Smartphone application |  |  |  |  |  |
| Leaflet or information sheet (e.g. Capsana, Kino Québec, etc.) |  |  |  |  |  |
| Other (specify) :  ______________________________ |  |  |  |  |  |

**16. When I intervene in the area of physical activity, I refer to:**_________________

[For each statement, check the one that represents your practice]

|  | Always | Often | Sometimes | Rarely | Never |
| --- | --- | --- | --- | --- | --- |
| A community activity (e.g. PIED program, Viactive, cadio plein air, etc.) |  |  |  |  |  |
| The kinesiologist from the CIUSSS MCQ Chronic Disease Prevention and Management program (e.g. risk factor clinic, Ma santé à vie, etc.) |  |  |  |  |  |
| The FMG kinesiologist |  |  |  |  |  |
| A private kinesiologist or physical activity specialist |  |  |  |  |  |
| Other (specify) :  ______________________________ |  |  |  |  |  |

**17. When I intervene through physical activity with individuals living with chronic diseases, I address the following elements:**
[For each statement, check what reflects your practice]

|  | Always | Often | Sometimes | Rarely | Never |
| --- | --- | --- | --- | --- | --- |
| Physical activity for leisure |  |  |  |  |  |
| Physical activity for travel purpose |  |  |  |  |  |
| Physical activity in work or main occupation |  |  |  |  |  |
| Physical activity for daily tasks |  |  |  |  |  |
| Number of physical activity sessions per week |  |  |  |  |  |
| Duration of each physical activity session |  |  |  |  |  |
| Intensity of physical activity sessions |  |  |  |  |  |
| Types of physical activity (walking, swimming, yoga, cycling, etc.) |  |  |  |  |  |
| Sedentary activity time |  |  |  |  |  |
| Other (specify) :  ______________________________ |  |  |  |  |  |

**18. Physical activity intervention for people with chronic diseases is important for**_______________? [For each statement, check the one that represents your practice]

|  | Strongly agree | Agree | Neither agree nor disagree | Disagree | Never |
| --- | --- | --- | --- | --- | --- |
|  |  |  |  |  |  |
| Increase daily energy expenditure |  |  |  |  |  |
| Increase overall muscle mass |  |  |  |  |  |
| Improve exercise tolerance |  |  |  |  |  |
| Prevent co-morbidities |  |  |  |  |  |
| Improve users' quality of life |  |  |  |  |  |
| Achieve clinical targets (e.g. lower blood pressure, reduce anxiety) |  |  |  |  |  |
| Improve self-management of the disease |  |  |  |  |  |
| Meet general physical activity recommendations (150 min. aerobic activity/week) |  |  |  |  |  |
| Other (specify) :  ______________________________ |  |  |  |  |  |

**19. The physical activity intervention is recorded in the user's file:**

Always

Often

Sometimes

Rarely

Never

**20.** **What are my limitations regarding to physical activity interventions for people with chronic diseases?** [For each statement, check the one that represents your opinion]

|  | Strongly agree | Agree | Neither agree nor disagree | Disagree | Never |
| --- | --- | --- | --- | --- | --- |
| Lack of time |  |  |  |  |  |
| Lack of knowledge of intervention tools and methods |  |  |  |  |  |
| Lack of knowledge of physical activity guidelines or best practices |  |  |  |  |  |
| Lack of knowledge about contraindications to physical activity |  |  |  |  |  |
| Lack of interest in physical activity on the part of users |  |  |  |  |  |
| Fear of harming the user |  |  |  |  |  |
| Colleagues' judgement of my intervention |  |  |  |  |  |
| Other (specify) : |  |  |  |  |  |

**21. What would help me optimize my physical activity interventions?** [For each statement, check the one that best represents your opinion]

|  | Strongly agree | Agree | Neither agree nor disagree | Disagree | Never |
| --- | --- | --- | --- | --- | --- |
| Training |  |  |  |  |  |
| Sharing of existing tools and methods |  |  |  |  |  |
| Clinical physical activity intervention tools (e.g. questionnaires, physical activity monitors) |  |  |  |  |  |
| Financial resources to purchase equipment |  |  |  |  |  |
| Access to clinical support (clinical expertise) |  |  |  |  |  |
| Other (specify) : |  |  |  |  |  |

| **Dimensions related to the implementation of an intervention** |
| --- |

**22. For each statement, check the option that best what represents your opinion.**

|  | Strongly agree | Agree | Neither agree nor disagree | Disagree | Never |
| --- | --- | --- | --- | --- | --- |
| I believe that regular physical activity contributes to users' health |  |  |  |  |  |
| I believe that physical inactivity is harmful to users' health. |  |  |  |  |  |
| I believe that healthcare professionals have a responsibility to promote physical activity to users. |  |  |  |  |  |
| I am confident in my ability to prescribe exercise. |  |  |  |  |  |
| I succeed in motivating users to start exercising. |  |  |  |  |  |
| I intend to prescribe physical activity more frequently to users. |  |  |  |  |  |

**23. At the FMG, I have access**________________________.
 [For each statement, check off what represents your situation]

|  | Strongly agree | Agree | Neither agree nor disagree | Disagree | Never |
| --- | --- | --- | --- | --- | --- |
| The establishment's intranet |  |  |  |  |  |
| The facility's clinical library |  |  |  |  |  |
| To the follow-up guidelines for various chronic diseases |  |  |  |  |  |
| Clinical support for physical activity |  |  |  |  |  |
| To continuing education on physical activity interventions |  |  |  |  |  |
| Other (specify) : |  |  |  |  |  |

**24.** **I consult the user's file to gather information on previous assessments physical activity interventions**:

Always

Often

Sometimes

Rarely

Never

**25. Interventions aimed at promoting the adoption of healthy lifestyle habits among users, including physical activity, are valued by** ____________:
[For each statement, check off what represents your opinion]

|  | Strongly agree | Agree | Neither agree nor disagree | Disagree | Never |
| --- | --- | --- | --- | --- | --- |
| The CIUSSS MCQ |  |  |  |  |  |
| My management/assistant management |  |  |  |  |  |
| My FMG |  |  |  |  |  |

**26. My clinic has been recognized as an FMG since :**

Less than a year

1 to 3 years

4 to 6 years

More than 6 years

Don't know

**27. To date, my FMG is recognized by the Ministry of Health and Social Services as a level**_______?

1 to 3

4 to 6

7 to 9

I don't know

How many non-physician professionals does the FMG have?

___________

**28. Over the past two years, I have participated in support and/or coaching activities (across various subjects) of the following types:**
[For each statement, check the one that best represents your situation]

|  | Strongly agree | Agree | Neither agree nor disagree | Disagree | Never |
| --- | --- | --- | --- | --- | --- |
| Co-development |  |  |  |  |  |
| Community of practice |  |  |  |  |  |
| Peer committee |  |  |  |  |  |
| Preceptorship/Mentorship |  |  |  |  |  |
| Clinical supervision |  |  |  |  |  |
| Other (specify) : |  |  |  |  |  |

**Section 2: Socio-demographic data**

**29. I am:**

Occupational therapist

Clinical nurse

Primary care nurse practitioner (PCNP)

Nurse

Kinesiologist

Physician

Attending physician

Nutritionist

Physiotherapist

Psychologist

Social worker

Others:________

**30. I am** _______________ **years old**

**31. My gender is**

Female

Male

Other:_____________

**32. My level of education is:**
 College
 University – Undergraduate (Bachelor’s degree or Medical Doctorate)
 University – Graduate (Master’s degree)
 University – Postgraduate (PhD)
 Other (specify): ________________

**33. My total number of years of experience is:** ___________________

**34. I have been working with individuals living with chronic diseases for:** ______ **years**

**35. My employment status is:**
 Full-time permanent
 Full-time temporary
 Part-time permanent
 Part-time temporary

**36. I practice in a Family Medicine Group (FMG):**
 Private (composed only of off-site locations)
 Public (composed only of on-site locations)
 Mixed (composed of both on-site and off-site locations)
 I don't know

**37. My clientele living with chronic diseases primarily resides in a:**
[Check the most appropriate answer]
☐ Rural area
☐ Urban area

**38. What percentage of your caseload consists of individuals with at least one chronic disease?**
____________ %

**39. Are there any other relevant data or information that should be considered but was not convered in this questionnaire?**

______________________________________________________________________

______________________________________________________________________

**40. Do you have any ideas or suggestions that could help enhance professional practices in the promoting physical activity? If so, please feel free to share them.**

______________________________________________________________________

______________________________________________________________________

**Acknowledgements**
The research team sincerely thanks you for the time and effort you have dedicated to this study. Your participation plays a vital role in advancing the quality of care for individuals living with chronic diseases. We deeply appreciate your contribution—thank you!
